# Multilevel mixed effects analysis of individual and community level factors associated with modern contraceptive use among married women in Uganda

**DOI:** 10.1186/s12889-021-11069-0

**Published:** 2021-07-27

**Authors:** Ronald Wasswa, Allen Kabagenyi, Paulino Ariho

**Affiliations:** 1 Department of Statistical Methods and Actuarial Science, School of Statistics & Planning, College of Business and Management Sciences, Makerere University, P.O. Box 7062, Kampala, Uganda; 2 Department of Population Studies, School of Statistics & Planning, College of Business and Management Sciences, Makerere University, P.O. Box 7062, Kampala, Uganda; 3 Department of Sociology and Social Administration, Kyambogo University, P.O. Box 1, Kampala, Uganda

**Keywords:** Modern contraceptives, Community, Married women, Uganda

## Abstract

**Background:**

In spite of the universal right for women to decide freely for themselves when, and how many children they want to have in life, married women in Uganda are less likely to use modern contraceptives as compared to other marital categories. This study examines the individual and community factors associated with modern contraceptive use among married women in Uganda.

**Methods:**

The study used data from the 2016 Uganda Demographic and Health Survey which comprised of 8671 married women aged 15–49 years who were fecund and non-pregnant at the time of the survey. Analysis was done using a multilevel mixed-effects logistic regression model.

**Results:**

Findings showed that married women who were; Muslims (AOR = 0.78, CI = 0.66–0.91), had more than five children (AOR = 0.76, CI = 0.61–0.98), staying in communities with high poverty (AOR = 0.78, CI = 0.65–0.93), with older age at first birth (AOR = 0.94, CI = 0.92–0.96) as well as having spousal age difference of more than 9 years (AOR = 0.86, CI = 0.76–0.98) were associated with low modern contraceptive use. Women living in communities with higher age at first marriage (AOR = 0.93, CI = 0.88–0.98) or higher sexual debut (AOR = 0.91, CI = 0.85–0.98) were also associated with reduced odds of modern contraception. In addition, older women (AOR = 1.03, CI = 1.01–1.04), having secondary/higher education (AOR = 1.93, CI = 1.58–2.37), living in a rich household (AOR = 1.32, CI = 1.14–1.53), short distance to health facility (AOR = 1.18, CI = 1.06–1.31), high community education (AOR = 1.38, CI = 1.17–1.62), high community exposure to family planning messages (AOR = 1.24, CI = 1.08–1.42), and communities with high proportion of women working (AOR = 1.22, CI = 1.06–1.39) were more likely to use modern contraceptives.

**Conclusion:**

The study revealed that both individual and community factors were important in explaining the factors associated with modern contraceptive use among married women in Uganda. Therefore, there is need to invest in community based programs like: family planning outreach services, mass media campaigns and community mobilization activities to help in dissemination of family planning information, increase awareness and promotion in use of modern contraceptives. Also, expansion of higher education and the need to make family planning services available and accessible to areas with limited physical access to health facilities will lead to sustained increase in uptake of modern contraceptives.

## Background

In 2019, it was estimated that only 41% of married women were using contraceptives globally [1]. In addition to demographic and health benefits to a woman, child, family and society, contraceptive use is an expression of a woman’s reproductive control and empowerment [2]. Non-use of modern contraception by women is attributed to lack of knowledge, limited access and choice of methods, high cost of contraceptives, fear of side-effects and cultural or religious or partner opposition [3, 4]. This has resulted into unplanned and early pregnancies, high birth rates which not only affect the mothers and their children but also increase government expenditure [5, 6].

Utilization of modern methods of contraceptives is noted to be lower in developing countries especially in sub-Saharan Africa (SSA) as compared to the developed countries [1, 7]. As a result of anticipated consequences of low contraceptive uptake, several governments have resorted to family planning campaigns with a view of delaying first births, lengthening birth intervals, reducing the total number of children born to a woman, preventing unintended pregnancies, better psychosocial well-being, reducing morbidity and mortality, better employment opportunities and education achievements among others [8]. Several studies on factors associated with use of contraceptives have been conducted in some developing countries like: Nepal, Zambia, Ethiopia, and Angola [9–12]. Most of the studies conducted concentrated on the influence of individual factors though few have also highlighted the impact of community factors on women’s use of contraceptives. Community education, community exposure to media [11]; place of residence [13–16]; region [11, 14, 15]; community employment [11]; community age at marriage and community age at sexual debut [17] are some of the important factors associated with contraceptive uptake.

Compared to neighboring countries like Tanzania, Rwanda and Kenya where contraceptive uptake was 35%, 35% and 48% respectively, the 2019 contraceptive uptake estimate for Uganda was 33% [1]. This has led to high unmet need for modern contraception and associated consequences [18]. In spite of the universal right for women to decide freely for themselves when, and how many children they want to have in life, married women in Uganda are less likely to use modern contraceptives compared to other marital categories [4, 13].

The 2016 UDHS reported that contraceptive prevalence rate among currently married women was much lower as compared to that of sexually active unmarried women at 39% and 51% respectively and 51% respectively [19]. Additionally, only 35% of the married women were using modern methods [19] which still remains very low to address the high rate of unintended pregnancies in Uganda. The proportion of women having mistimed births or current pregnancies increased from 21% in 1995 to 32% in 2016 whereas that of unwanted births or pregnancies slightly rose from 8% in 1995 to 9% in 2016. Among currently married women in Uganda, the total demand for family planning increased from 54% in 2000–01 to 67% in 2016 but only 52% of demand was satisfied by modern methods [19].

Studies [20–23] on contraceptive uptake in Uganda have been conducted but these do not evaluate whether modern contraceptive use can be attributed to individual differences or differences within their communities. It is thus important in this study, to examine both individual and community factors associated with modern contraceptive use among married women so that appropriate interventions can be made to improve the use of contraceptives.

## Methods

### Data source

**Fig. 1:**
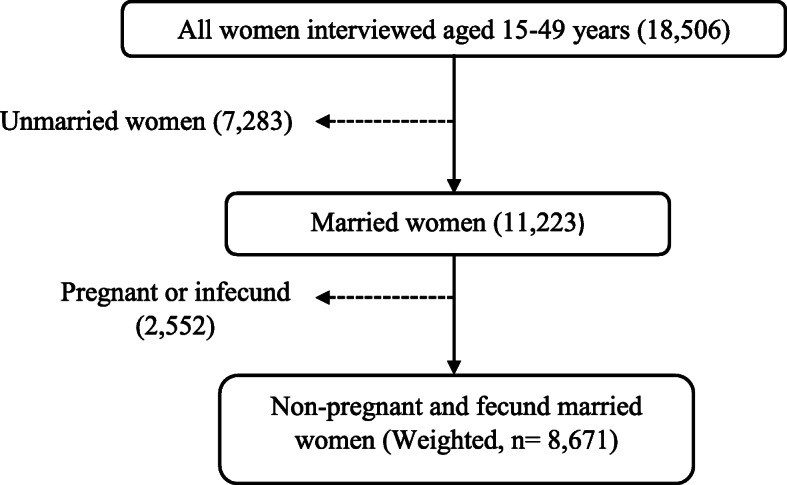
Sample selection procedure

Data for this study were obtained from the 2016 Uganda Demographic and Health Survey (UDHS). The 2016 UDHS was a national representative survey that employed a two stage stratified sample design. In the first stage, 696 enumeration areas (EAs) were selected from a list of clusters based on the 2014 Uganda Population and Housing Census sample frame [19]. In Uganda, an enumeration area is a geographical area that covers an average of 130 households [19]. These EAs were chosen independently basing on probability proportional to size. The second stage involved a systematic sampling of 20,880 households within each cluster from which all women of child bearing age (15–49 years), who were either permanent residents of the households or visitors who slept in the households the night before the survey were eligible to be interviewed. A total of 18,506 women aged 15–49 years were interviewed. In this study, women who were never married as well as those who were divorced, separated, widowed were excluded. Also, currently married women who were pregnant as well as those who reported that they were declared infecund were excluded. Therefore, the final study sample included a weighted total of 8671 non-pregnant and fecund married women. Figure 1 shows the derivation of the study sample.

### Variables and their measurements

In this study, modern contraceptive use was the dependent variable. The 2016 UDHS defined modern contraceptive use as, the use by a woman or her partner of at least one of the following methods: male or female sterilization, injectable, intrauterine devices, contraceptive pills, implants, female or male condoms, standard days method, lactational amenorrhoea, and emergency contraception [19]. Based on this definition, we generated modern contraceptive use as a binary outcome which was coded 1 if a woman was currently using a modern contraceptive method at the time of the survey and 0 if a woman was either using a traditional method or not using any method. Traditional methods included the use of rhythm, periodic abstinence, withdrawal, and folk methods.

The independent variables were categorized into two: individual and community factors. Individual factors included woman’s age (15–24, 25–34, and 35–49), level of education attainment (no education, primary, secondary/higher), spousal age difference generated from the information provided by women on their current age and that of their husbands categorized as (0–4, 5–9 and ≥ 10); wealth index (The UDHS generates wealth index using principal component analyses applied on a number and kinds of consumer goods a household owns, ranging from a television to a bicycle or car, and housing characteristics such as source of drinking water, toilet facilities, and flooring materials. From this, five quintiles of wealth index (lowest, second, middle, fourth and highest) are generated with the first quintile representing 20% poorest households, and the last quintile corresponding to 20% wealthiest (richest) households). In this study, wealth index constituted three categories, that is: poor (merging lowest and second), middle, and rich (merging fourth and highest quintiles).

Other variables were: working status (currently working and not working); religion (Catholic, Anglicans, Muslims, Others; where other religions include Seventh Day Adventists (SDA), Orthodox, Born again/Pentecostal/Evangelical, Baha’i, Baptist, Presbyterian, Jehovah’s witness, Salvation army, Traditionalists and other unknown religions); age at first birth (< 20 and **≥** 20); parity (0–2, 3–5 and 6+); women’s empowerment (this was based on three questions as stated in the UDHS: a person who usually decides on respondent’s health care; a person who usually decides on large household purchases; a person who usually decides on visits to family or relatives). In this study, a woman was considered to be empowered if she could decide alone and make independent decisions regarding her own healthcare. We did this by recoding the response categories to the three questions as: yes = 1 for decisions made by a woman alone and no = 0 for all decisions made by either the husband or a joint decision. A binary variable of woman’s empowerment was then generated with a woman who had at least 2 out of the 3 decisions made by herself considered to be empowered (coded yes = 1) while a woman who had less than 2 out of 3 decisions made by herself was considered not to be empowered (coded no = 0). This approach was considered to be appropriate and has been applied elsewhere [24]. Exposure to family planning (FP) messages was another variable measured in the 2016 UDHS using four questions that required whether a woman was exposed to family planning messages on radio, television, newspapers and phone in the last month before the survey. All the responses to these questions were merged and coded 0 for no exposure to all the media and 1 for those who heard FP messages from any of the four sources.

Community factors included: place of residence (urban, rural); region (Central, Eastern, Western, and Northern); Distance to health facility: in the 2016 UDHS, women were asked if the distance to the health facility was a problem for them to get any medical assistance and responses were coded as: 0 if it was big problem and 1 if it was not a big problem. This study applied the same codes. Other community factors were obtained by aggregation of individual level characteristics of women within their clusters (communities) since the 2016 UDHS did not directly capture data that could describe these characteristics and the average values at the cluster level used as the cut off point for the categorization. In this case, each woman was assigned a value representing the average response of all other respondents in her cluster. These factors included: community education (coded as low for values below the average level of education while high for values equal or above the mean); community poverty (coded as low for values equal and above the mean basing on the wealth index categories of the individual women while high for values below the mean); community exposure to family planning (FP) messages (coded as low for values below the mean while high for values equal or above the mean); community women working (coded as low for values below the mean while high for values equal or above the mean). Other community variables included community mean age at first marriage and community mean age at sexual debut which were treated as continuous variables.

### Data analysis

Data analysis was done at three stages using STATA statistical software. Sampling weights provided in the DHS data were applied in order to adjust for the sampling design. At the first stage of analysis, we generated a descriptive summary of the variables using frequency distributions for categorical variables and mean values for continuous variables. At the second stage, we used a bivariate multilevel logistic regression to examine the crude association between the individual and community-level factors with modern contraceptive uptake and a *p*-value < 0.05 set as a significant level at 95% confidence level. At the third stage; a multivariate two-level mixed-effects logistic regression model was applied to investigate the impact of the community factors on the outcome variable with married women at level 1 being nested within communities at level 2. The analysis at this stage took four steps using four models: Model 1 (Empty model) was fitted without explanatory variables to test the random variability in the intercept and show the total variance in the use of modern contraceptives among women in different communities. Model 2, examined the effect of individual level characteristics. Model 3 examined the effect of community level variables and Model 4 examined the effect of both individual and community level characteristics simultaneously with the results of fixed effects being shown as odds ratios at 95% confidence level. The Inter-cluster correlation coefficient (ICC) for each model was calculated to explain the proportion of variation that was attributable to the higher level of variation and to compare the successive models. These values were obtained from, $\mathrm{ICC}=\frac{\sigma^2}{\left({\sigma}^2+\frac{\pi^2}{3}\right)}$ where *σ*^2^ is the estimated community level variance while $\frac{\pi^2}{3}$ is the household variance. The Proportional Change in Variance (PCV) was also computed for each model with respect to the empty model to show the power of the factors in the models in explaining the outcome variable. The PCV was obtained from $PCV=\left(\frac{V_e-{V}_i}{V_e}\right)$ where V_e_ is variance of modern contraceptive use in the empty model and V_*i*_ is variance in successive models. The two-level multilevel model with a binary response variable for a married woman *i* living in community *j*, is represented as:

$$ \mathit{\log}\left[\frac{\pi_{ij}}{1-{\pi}_{ij}}\right]={\beta}_0+{\beta}_1{X}_{1 ij}+\dots +{\beta}_n{X}_{nij}+{\upsilon}_{0j}+{\varepsilon}_{ij} $$

Where: *π*_*ij*_ is the probability that the *i*^th^ married woman in the *j*^th^ community was currently using a modern contraceptive; (1 − *π*_*ij*_) is the probability that she was not using a modern contraceptive; *β*_0_ is the log odds of the intercept; *β*_1_, *β*_2_, …, *β*_*n*_ are the effect sizes by the individual and community-level variables; *X*_1*ij*_, *X*_2*ij*_, …*X*_*nij*_ are the independent variables at individual and community levels; *υ*_0*j*_ and *ε*_*ij*_ are random errors at community and individual levels respectively.

## Results

Table 1 presents the selected individual characteristics of currently married women aged 15–49 years who were nested within 696 communities with the number of women per community ranging from 3 to 25 (average 13). The results indicate that the average age of married women in the study was 30 years with 41% aged 25–34 years. The table further shows that only 45% of the married women were using modern contraceptives, 39% were Catholics, 59% had a primary level of education, 4 in 10 were from poor households, 80% were working and 66% had their first birth before 20 years with the average age at first birth being 19 years. Additionally, 45% of married women had a spousal age difference of less than 5 years, only 20% were empowered, 29% had more than 5 children while 70% had access to family planning messages through media.

**Table 1:** Distribution of married women by background characteristics

Individual characteristic	Number (8671)	Percentage (%)
Woman’s age	8671	*30.2(7.9)*
15–24	2476	28.6
25–34	3564	41.1
35–49	2631	30.3
Religion		
Catholic	3420	39.4
Anglican	2714	31.3
Muslim	1178	13.6
Other	1359	15.7
Level of educational attainment		
No education	940	10.8
Primary	5134	59.2
Secondary/Higher	2597	30.0
Wealth index		
Poor	3300	38.1
Middle	1674	19.3
Rich	3697	42.6
Working status		
Not working	1713	19.8
Currently working	6958	80.2
Spousal age difference		
0–4	3937	45.4
5–9	2890	33.3
≥ 10	1844	21.3
Age at first birth	8361	*18.7(3.2)*
< 20	5507	65.9
≥ 20	2854	34.1
Parity		
0–2	2837	32.7
3–5	3340	38.5
6+	2494	28.8
Woman’s empowerment		
No	6962	80.3
Yes	1709	19.7
Media exposure on family planning messages		
No	2590	29.9
Yes	6081	70.1
Modern contraceptive use		
No	4770	55.0
Yes	3901	45.0

Italic items in the percentage column indicate mean (SD) respectively**.** SD is the standard deviation with all estimates based on weighted data

Table 2 indicates the distribution of women by selected community characteristics. The table shows that three quarters (76%) of the married women lived in rural areas while slightly more than a quarter (28%) were from Central region. The results in the table also show that 38% married women highlighted that the distance to the health facility was a big problem while more than half of women lived in communities that had a high proportion of: low education (51%), low poverty (53%), high access to family planning messages (54%) and highly working women (53%). Further, the community mean age at first marriage and mean age at sexual debut were 18 and 17 years respectively.

**Table 2:** Distribution of married women by community characteristics

Community characteristic	Number (8671)	Percentage (%)
Place of residence		
Urban	2107	24.3
Rural	6564	75.7
Region		
Central	2395	27.6
Eastern	2349	27.1
Western	2273	26.2
Northern	1654	19.1
Distance to health facility		
Big problem	3330	38.4
Not a big problem	5341	61.6
Community education		
Low	4374	50.5
High	4297	49.5
Community poverty		
Low	4584	52.9
High	4087	47.1
Community exposure to family planning messages		
Low	3953	45.6
High	4718	54.4
Community women working		
Low	4059	46.8
High	4612	53.2
Community mean age at first marriage(mean, SD)	8671	*18.4(1.6)*
Community mean age at sexual debut(mean, SD)	8671	*16.6(1.1)*

Italic items in the percentage column indicate mean (SD) respectively**.** SD is the standard deviation with all estimates based on weighted data

### Modern contraceptive uptake among individuals and communities

The unadjusted odds ratio results in Table 3 indicate that married women aged 25–34 or 35–49 years, Anglican, those with primary or secondary/higher education, women who were currently working, those living in rich households and women with exposure to mass media family planning messages had increased odds of modern contraceptive uptake. Relatedly, compared to those whose spousal age difference was 0–4 years or those with parity of 0–2 children, women with a spousal age difference of 5–9 years or parity of 3–5 as well as 6+ parity were more likely to be using modern contraceptives at the time of the survey. Table 3 also indicates that married women in rural areas, those from eastern or northern regions were associated with lower likelihood of modern contraceptive use.

**Table 3:** Association of modern contraceptive use with the selected characteristics

Characteristic	OR(95% CI)
Woman’s age	1.02(1.02–1.03)**
15–24^a^	1.00
25–34	1.50(1.31–1.71)**
35–49	1.61(1.41–1.81)**
Religion	
Catholic^a^	1.00
Anglican	1.18(1.06–1.33)**
Muslim	0.94(0.80–1.10)
Other	0.93(0.81–1.08)
Level of educational attainment	
No education^a^	1.00
Primary	1.54(1.32–1.81)**
Secondary/Higher	1.85(1.55–2.20)**
Wealth index	
Poor^a^	1.00
Middle	1.48(1.30–1.69)**
Rich	1.78(1.58–2.00)**
Working status	
Not working^a^	1.00
currently working	1.27(1.13–1.43)**
Spousal age difference	
0–4^a^	1.00
5–9	1.15(1.04–1.28)**
≥ 10	0.95(0.84–1.07)
Age at first birth	0.96(0.94–0.97)**
< 20^a^	1.00
≥ 20	0.75(0.68–0.83)**
Parity	
0–2^a^	1.00
3–5	1.48(1.32–1.65)**
6+	1.42(1.26–1.60)**
Woman’s empowerment	
No^a^	1.00
Yes	1.02(0.91–1.15)
Media exposure to family planning messages	
No^a^	1.00
Yes	1.25(1.13–1.38)**
Place of residence	
Urban^a^	1.00
Rural	0.73(0.62–0.85)**
Region	
Central^a^	1.00
Eastern	0.77(0.64–0.92)**
Western	0.85(0.71–1.02)
Northern	0.50(0.41–0.61)**
Distance to health facility	
Big problem^a^	1.00
Not a big problem	1.26(1.14–1.39)**
Community education	
Low^a^	1.00
High	1.78(1.56–2.02)**
Community poverty	
Low^a^	1.00
High	0.54(0.47–0.61)**
Community exposure to family planning messages	
Low^a^	1.00
High	1.54(1.35–1.76)**
Community women working	
Low^a^	1.00
High	1.04(0.91–1.19)
Community mean age at first marriage	1.01(0.97–1.05)
Community mean age at sexual debut	0.97(0.92–1.03)

^a^ is a Reference category; OR is the unadjusted odds ratio; 95% CI is the Confidence Interval; *p < 0.05, **p < 0.01; the assessment was based on bivariate-multilevel logistic regression model with  χ^2^ < 0.001

Further, in regards to community factors, communities in which distance to a health facility was not a big problem to access health care as well as those with; higher education, lower poverty, and higher media exposure family planning messages were highly associated with higher uptake of modern contraceptives.

### Individual and community characteristics associated with modern contraceptive uptake

Table 4 shows results of four models in the uptake of modern contraceptives by married women. Model 1 had only the dependent variable with the results showing a statistically significant variability in the odds of modern contraceptive use between communities (τ = 0.39, *p*-value < 0.01). The ICC in this model indicated that 11% of the total variance in the modern contraceptive use was attributed to differences between communities. In model 2, individual level variables were included. The results showed that; woman’s age, religion, wealth index, level of education attainment, spousal age difference, parity and age at first birth were significantly associated with modern contraceptive use. The ICC in this model indicated that 7.5% of the variation in modern contraceptive use was attributed to differences across communities where as a PCV implied that 31% of the variance in modern contraceptive use across communities was explained by these individual characteristics. In Model 3, only community level variables were added and the results revealed that women residing in communities with high media exposure to family planning message, high education level and high proportion of women working were positively associated with modern contraceptive use while married women from the Northern region, those residing in communities with high poverty and those in communities with a higher age at first marriage and higher age at sexual debut had an inverse relationship. The ICC in this model showed that differences between communities account for 7% of the variation in modern contraceptive use while the PCV indicated that 39% of the community variation in modern contraceptive use was explained by community level characteristics.

**Table 4:** Multilevel mixed effect analysis of individual and community level factors associated with modern contraceptive use among married women

Characteristic	Model 1 Empty	Model 2 AOR(95% CI)	Model 3 AOR(95% CI)	Model 4 AOR(95% CI)
Woman’s age		1.03(1.01–1.04)**		1.03(1.01–1.04)**
15–24^a^		1.00		1.00
25–34		1.30(1.09–1.55)**		1.30(1.09–1.55)**
35–49		1.26(0.92–1.74)		1.26(0.91–1.73)
Religion				
Catholic^a^		1.00		1.00
Anglican		1.13(1.01–1.27)*		1.09(0.97–1.22)
Muslim		0.83(0.70–0.97)*		0.78(0.66–0.91)**
Other		0.91(0.79–1.05)		0.90(0.78–1.04)
Level of educational attainment				
No education^a^		1.00		1.00
Primary		1.63(1.38–1.93)**		1.50(1.27–1.78)**
Secondary/Higher		2.17(1.78–2.64)**		1.93(1.58–2.37)**
Wealth index				
Poor^a^		1.00		1.00
Middle		1.42(1.24–1.63)**		1.27(1.10–1.46)**
Rich		1.61(1.41–1.83)**		1.32(1.14–1.53)**
Working status				
Not working^a^		1.00		1.00
currently working		1.15(1.02–1.31)*		1.13(0.99–1.28)
Spousal age difference				
0–4^a^		1.00		1.00
5–9		1.08(0.97–1.21)		1.07(0.96–1.19)
≥ 10		0.87(0.76–0.98)*		0.86(0.76–0.98)*
Age at first birth		0.94(0.91–0.96)**		0.94(0.92–0.96)**
< 20^a^		1.00		1.00
≥ 20		0.81(0.69–0.95)*		0.82(0.70–0.96)*
Parity				
0–2^a^		1.00		1.00
3–5		0.93(0.80–1.07)		0.94(0.81–1.09)
6+		0.72(0.58–0.90)**		0.76(0.61–0.94)*
Woman’s empowerment				
No^a^		1.00		1.00
Yes		0.94(0.84–1.06)		0.95(0.84–1.07)
Media exposure to family planning messages				
No^a^		1.00		1.00
Yes		1.08(0.97–1.20)		1.03(0.92–1.15)
Place of residence				
Urban^a^			1.00	1.00
Rural			0.92(0.78–1.09)	0.94(0.79–1.11)
Region				
Central^a^			1.00	1.00
Eastern			0.87(0.72–1.05)	0.88(0.73–1.07)
Western			1.06(0.89–1.27)	1.08(0.90–1.29)
Northern			0.75(0.61–0.93)**	0.83(0.67–1.04)
Distance to health facility				
Big problem^a^			1.00	1.00
Not a big problem			1.16(1.04–1.28)**	1.18(1.06–1.31)**
Community education				
Low^a^			1.00	1.00
High			1.52(1.30–1.78)**	1.38(1.17–1.62)**
Community poverty				
Low^a^			1.00	1.00
High			0.66(0.56–0.79)**	0.78(0.65–0.93)**
Community exposure to family planning messages				
Low^a^			1.00	1.00
High			1.28(1.12–1.46)**	1.24(1.08–1.42)**
Community women working				
Low^a^			1.00	1.00
High			1.31(1.15–1.49)**	1.22(1.06–1.39)**
Community mean age at first marriage			0.92(0.87–0.96)**	0.93(0.88–0.98)**
Community mean age at sexual debut			0.89(0.82–0.95)**	0.91(0.85–0.98)**
Random effect				
Community level variance (SE)	0.39(0.04)**	0.27(0.04)**	0.24(0.03)**	0.22(0.03)**
ICC (%)	10.7	7.5	6.8	6.3
PCV (%)	Reference	30.8	38.5	43.6
Model fit statistics				
Log likelihood	− 5822	− 5466	− 5726	-5419
AIC	11,648	10,973	11,477	10,902

^a^ is a Reference category, * Significant at *p* < 0.05, ** significant at *p* < 0.01; AOR is the adjusted odds ratio; SE is the standard error; ICC is the inter-cluster correlation coefficient; PCV is the Proportional Change in Variance

Model 4 included both the individual and community level variables. In this model, the variation in the odds of modern contraceptive use between communities remained statistically significant (τ = 0.22, *p*-value < 0.001) with an estimated 6% of the variability in modern contraceptive use attributed to differences between communities and 44% of the variation in modern contraceptive use across communities being explained by both individual and community level factors. The results in model 4 show that an increase in woman’s age increases the odds of modern contraceptive uptake (AOR = 1.03, CI = 1.01–1.04). Specifically, women aged 25–34 years were more likely (AOR = 1.30, CI = 1.09–1.55) to use modern contraceptives compared to those less than 25 years. The results also indicate that married Muslim women were less likely (AOR = 0.78, CI = 0.66–0.91) to use modern contraceptives relative to Catholics. Women with primary education (AOR = 1.50, CI = 1.27–1.78) or secondary/higher education (AOR = 1.93, CI = 1.58–2.37), those from middle (AOR = 1.27, CI = 1.10–1.46) or rich households (AOR = 1.32, CI = 1.14–1.53) had increased odds of using modern contraceptives. The findings also indicate that couples whose age difference was at least 10 years (AOR = 0.86, CI = 0.76–0.98) as well as women with more than 5 children (AOR = 0.76, CI = 0.61–0.94) were less likely to use modern contraceptives. Still, increasing age at first birth lowers the odds of using modern contraceptive (AOR = 0.94, CI = 0.92–0.96) with those that had their first birth after 19 years being less likely (AOR = 0.82, CI = 0.70–0.96) compared to those whose first births were before 19 years.

Regarding community effect, married women who reported that the distance to the health facility was not a big problem were more likely (AOR = 1.18, CI = 1.06–1.31) to use modern contraceptive methods compared to their counterparts who reported otherwise. The results in Table 4 also indicate that married women living in communities with high education levels (AOR = 1.38, CI = 1.17–1.62), high working levels (AOR = 1.22, CI = 1.06–1.39) and those in communities with high exposure to family planning messages (AOR = 1.24, CI = 1.08–1.42) were more likely to use modern contraceptive methods. However, the results still revealed that married women living in communities with high poverty (AOR = 0.78, CI = 0.65–0.93), women living in communities with higher mean age at first marriage (AOR = 0.93, CI = 0.88–0.98) and those in communities with higher age at sexual debut (AOR = 0.91, CI = 0.85–0.98) were associated with lower odds for modern contraceptive uptake.

## Discussion

In this study, we analyzed individual and community factors associated with modern contraceptive use among married women aged 15–49 years in Uganda. Our findings revealed that both individual and community level factors were equally important in explaining the factors associated with modern contraceptive use among married women in Uganda. Results also showed that, woman’s age, religion, wealth index, level of education attainment, spousal age difference, age at first birth, parity, distance to health facility, community education, community poverty, community exposure to family planning messages, community working status, community mean age at first marriage and community mean age at first sex debut had a significant influence on modern contraceptive use among married women.

Our results indicated that older women were more likely to use modern contraceptives as compared to the young ones. This could be attributed to the fact that older married women are assumed to be knowledgeable and confident about different modern contraceptive methods that may suit their needs as compared to their young counterparts. The finding could also be because older women have achieved their desired fertility or have had some children and thus desire to either stop or space their next births compared to the young women who are likely to be in their earlier years of marital unions and thus under pressure to have children as soon as possible. This finding partly concurs with previous studies [9, 25].

Modern contraceptive use also decreases with increasing age gap between couples. More specifically, married women whose age difference with their partners was more than 9 years were less likely to use modern contraceptives compared to those couples with an age difference of less than 5 years. This outcome is consistent with studies conducted in Nigeria [26], Zambia [10], and Tanzania [27]. Similarly, a study conducted by Magali and colleagues [28] on the impact of age difference between spouses and contraceptive practice in sub-Saharan Africa revealed a positive correlation between modern contraceptive use and couples’ small age difference. Women whose partners are at least 10 years older tend to have low individual decision making power and low marital bond [28] and this could partly explain their low uptake for contraception in this study.

Results also indicate that Muslim married women were less likely to use modern contraceptives compared to Catholics. This was in line with other findings such as those in Ethiopia [11] and Nigeria [16, 28]. However, this finding does not relate to the known doctrines in the Catholic [29, 30] and Islamic [31] faiths. Therefore, there is need for further investigation on this religious disparity in modern contraceptive uptake among married women in Uganda.

In regard to education, educated women were more likely to use modern methods of contraception compared to uneducated ones. The possible explanation for this could be that education empowers women with information, enhances access to resources that may be used to access contraceptives and also increases their bargaining power and participation in decision making processes. Our finding partly concurred with other studies [9, 15, 16, 32, 33].

We also found that women living in communities characterized by high education were more likely to use modern contraceptives relative to those who lived in communities with low education and this correlated with earlier findings [22]. Therefore, educating a community helps in shaping the health seeking behaviors of its individuals including knowledge and easy access of family planning.

The findings also indicate that the wealth of the household is an important factor in women’s use of modern family planning methods. Women in the middle or rich households had higher odds of modern contraceptive use compared to their counterparts in the poor households. This is because women from wealthier households may have better access to modern contraceptives as compared to those from poor households. This finding corroborates with the findings that have been reported in previous studies [13, 32–34].

The study also shows that married women with more than 5 children were less likely to use modern contraceptive as compared to those with fewer children. Our finding was inconsistent with other findings in Nepal [9], Afghanistan [33] and Angola [12]. The possible reason for this disparity may be due to the fact that, high parity women may not have the knowledge on the proper use of modern contraceptives and may also have low access to family planning services.

Our findings still show that women who indicated that accessing health care facilities was not a big problem were more likely to use modern contraceptive methods compared to those who revealed problem challenges. This finding is in line with earlier studies [35–37]. Physical proximity to health facilities plays an important role in the health-seeking behavior like access of modern contraceptives in a community [35]. Long distances therefore demotivate women who may want to consistently use family planning services which later result to discontinuation and intermittent use [36]. This finding highlights the need to make family planning services available and accessible in all parts of the country.

Findings also revealed that married women living in communities with high poverty levels were less likely to use modern contraceptives compared to those who lived in communities that were in low poverty concurring with other findings [11]. The possible explanation could be that, economically poor communities may not invest in women education thus have low knowledge on contraceptive use, low autonomy and the distance to health facilities may also be a big problem making the uptake of contraceptives very low.

Furthermore, communities that have more working married women had higher odds of modern contraceptives and this was in line with the findings in Ethiopia [11]. Women who are working are able and willing to meet the financial health costs required and are also empowered in making decisions with their partners regarding family planning which may not be the case with married women who are not working and are entirely dependent on their husbands.

Though the association between individual access to family planning messages and modern contraceptive uptake by married women was insignificant, we find that community exposure to family planning messages on the other hand was very important. Married women in communities that have high exposure to family planning messages were more likely to use modern contraceptives compared to those living in communities that have low exposure. This finding suggests that mass media campaigns and community mobilization activities can help in increasing awareness and promoting the use of modern contraceptives among women. This was in agreement with the findings across Europe [17].

Married women who were living in communities with a higher mean age at first marriage and those in communities with higher age at sexual debut at the time of the 2016 UDHS were less likely to use modern contraceptives. This finding is in agreement with the findings of Mutumba et al. among Americans [17]. Other studies have also found that age at first marriage and sexual debut are positively associated with age at first birth and inversely associated with desired fertility [37–39]. This is partly because a rise in community age at first marriage and community age at first sex may influence women within the communities to postpone their time to first births and these are likely to bear their first children soon after marriage not only to compensate for their late start but also achieve their desired fertility just like their peers who initiated marriage and sex earlier. Therefore, such married women may not use modern contraceptives.

Relatedly, we found that an increase in age at first birth, lowers the use of modern contraceptives as women who had their first birth before 20 years were more likely to use modern contraceptives compared to those whose first births were after 20 years. This concurred with the findings of Mutumba et al., among Africans and European countries [17]. Previous findings by Wasswa and colleagues revealed that married women in Uganda with higher age at first birth were at an increased risk of unintended pregnancies [40]. In the process of wanting to achieve their desired number of children, women with higher age at first birth may avoid using contraceptives.

### Strengths and limitations of the study

The strength of this manuscript is that it is based on a nationally representative sample of married women. Also, the analytical approach identifies the contribution of individual and community characteristics as well as exploring the variation in modern contraceptive uptake that can be linked to community and individual differences. However, being a cross-sectional study, we are unable to determine causation. Never the less, our findings show the individual and community factors associated with modern contraceptive use among married women in Uganda as well as the variations in modern contraceptive uptake that are associated with community differences. These findings are thus important for formulation of community tailored interventions that may result in increased uptake of modern contraceptive methods. The study is also based on a quantitative survey and thus misses on the qualitative individual and community aspects surrounding modern contraceptive uptake. These can be examined by future studies.

## Conclusion

This study revealed that about 45% of the married women were using modern contraceptives. Both individual and community factors were important in explaining modern contraceptive use among married women in Uganda. We find that an estimated 6% of the variability in modern contraceptive use may be attributed to differences between communities and 44% of the variation in modern contraceptive use across communities is due to individual and community level factors.

The key individual and community factors associated with low modern contraceptive uptake were: being uneducated, young married women, Muslim women, the poor, spousal age difference of more than 10 years, women burdened with higher fertility, older age at first birth, long distance to health facility and low community education. Others were: high community poverty, low community exposure to family planning messages, low proportion of women working in a community, higher community age at marriage and higher community age at sexual debut were all associated with low modern contraceptive use.

The government should invest in community based programs such as: family planning outreach services, mass media campaigns and community mobilization activities in order to help in dissemination of family planning information, increase awareness and promote the use of modern contraceptives. Still, expansion of higher education among women and the need to make family planning services available and accessible to areas with limited physical access to health facilities will also lead to sustained increase in uptake of modern contraceptives.

## Data Availability

The dataset used and analyzed in this paper is available from the DHS program site upon request: http://dhsprogram.com/data/

## Abbreviations

AOR: Adjusted odds ratios
CI: Confidence interval
FP: Family Planning
ICC: Intra cluster correlation
OR: Odds ratio
PCV: Proportional change in variance
UBOS: Uganda Bureau of Statistics
UDHS: Uganda Demographic and Health Survey
WHO: World Health Organization
